# An Enzybiotic Regimen for the Treatment of Methicillin-Resistant *Staphylococcus aureus* Orthopaedic Device-Related Infection

**DOI:** 10.3390/antibiotics10101186

**Published:** 2021-09-29

**Authors:** Eric T. Sumrall, Marloes I. Hofstee, Daniel Arens, Christian Röhrig, Susanne Baertl, Dominic Gehweiler, Mathias Schmelcher, Martin J. Loessner, Stephan Zeiter, R. Geoff Richards, T. Fintan Moriarty

**Affiliations:** 1 AO Research Institute Davos, 7270 Davos, Switzerland; eric.sumrall@aofoundation.org (E.T.S.); marloes.hofstee@aofoundation.org (M.I.H.); daniel.arens@aofoundation.org (D.A.); susanne.baertl@ukr.de (S.B.); dominic.gehweiler@aofoundation.org (D.G.); stephan.zeiter@aofoundation.org (S.Z.); geoff.richards@aofoundation.org (R.G.R.); 2 Micreos GmbH, 8820 Wädenswil, Switzerland; c.roehrig@micreos.com (C.R.); mathias.schmelcher@hest.ethz.ch (M.S.); 3 Department of Trauma Surgery, University Medical Center, 93053 Regensburg, Germany; 4 Institute of Food, Nutrition and Health, ETH Zürich, 8092 Zurich, Switzerland; martin.loessner@ethz.ch

**Keywords:** *Staphylococcus aureus*, MRSA, biofilm, orthopaedic infection, osteomyelitis, fracture-related infection, enzybiotic, endolysin

## Abstract

Orthopaedic device-related infection (ODRI) presents a significant challenge to the field of orthopaedic and trauma surgery. Despite extensive treatment involving surgical debridement and prolonged antibiotic therapy, outcomes remain poor. This is largely due to the unique abilities of *Staphylococcus aureus*, the most common causative agent of ODRI, to establish and protect itself within the host by forming biofilms on implanted devices and staphylococcal abscess communities (SACs). There is a need for novel antimicrobials that can readily target such features. Enzybiotics are a class of antimicrobial enzymes derived from bacteria and bacteriophages, which function by enzymatically degrading bacterial polymers essential to bacterial survival or biofilm formation. Here, we apply an enzybiotic-based combination regimen to a set of in vitro models as well as in a murine ODRI model to evaluate their usefulness in eradicating established *S. aureus* infection, compared to classical antibiotics. We show that two chimeric endolysins previously selected for their functional efficacy in human serum in combination with a polysaccharide depolymerase reduce bacterial CFU numbers 10,000-fold in a peg model and in an implant model of biofilm. The enzyme combination also completely eradicates *S. aureus* in a SAC in vitro model where classical antibiotics are ineffective. In an in vivo ODRI model in mice, the antibiofilm effects of this enzyme regimen are further enhanced when combined with a classical gentamicin/vancomycin treatment. In a mouse model of methicillin-resistant *S. aureus* (MRSA) ODRI following a fracture repair, a combined local enzybiotic/antibiotic treatment regimen showed a significant CFU reduction in the device and the surrounding soft tissue, as well as significant prevention of weight loss. These outcomes were superior to treatment with antibiotics alone. Overall, this study demonstrates that the addition of enzybiotics, which are distinguished by their extremely rapid killing efficacy and antibiofilm activities, can enhance the treatment of severe MRSA ODRI.

## 1. Introduction

Orthopaedic device-related infections (ODRI) are some of the most devastating complications in modern orthopaedic and trauma surgery [1], with an incidence rate of up to 25% following treatment of an open fracture [2,3]. Treatment typically involves surgical debridement of necrotic or infected hard and soft tissue and application of local antibiotics as an adjunct to prolonged systemic antibiotic therapy [3]. Despite current treatment strategies, long-term outcomes remain poor [4] and are further burdened by significant socioeconomic costs [5]. The goal when treating ODRI is not only to address the established infection but to minimize the impact on the patient [6]. Surgical interventions follow one of two possible principles: device retention or removal and replacement. Retaining the infected device is always preferred, as it requires fewer surgical procedures; however, it is not suitable for all cases. Device retention is possible if the infection is at an early stage, overlying soft tissues are in good condition, the fracture is stable, and the pathogen is not highly resistant to key antibiotics [3]. Device stability is particularly important since instability has been demonstrated to be an independent risk factor for failure [7] and has long been considered important in bone healing [8]. Recent studies have found infection treatment success rates with device retention to be only 70–75% [9,10,11,12,13]. Thus, novel interventions that may increase treatment success rates and enable device retention have great potential for improving the care of patients with ODRI.

*Staphylococcus* sp. is the most frequently isolated causal agent of bone-related infection [14] at a rate of ~66% [15]. The increasing prevalence of methicillin-resistant *S. aureus* is also contributing to the severity of the problem [16]. Orthopaedic infection is usually a result of bacterial communities growing as biofilms either directly on an implanted device or within the protective niches of necrotic bone tissue [17]. Existing in over 60% of all chronically infected fracture wounds [18], biofilms are perhaps one of the most important weapons bacteria hold in their arsenal that must be overcome during treatment. Bacteria residing as biofilms in the bone niche or on a foreign device are protected from both the immune system as well as antimicrobials by forming a protective matrix. This matrix can contain extracellular polysaccharides (EPS), teichoic acids, DNA, lipids, and proteins. Besides protecting the bacterial community from its dangerous environment [19,20], these EPS can serve as a platform to mediate adhesion to bone and foreign surfaces such as implanted devices [19]. Some bacteria in biofilms can be metabolically inactive, making them difficult to identify, culture, and treat [21,22,23]. These so-called “persister cells” can maintain complete resistance to certain antibiotics [24,25], and their exit from a dormant into an active state is thought to cause recurrent infection [26]. *S. aureus* can also form staphylococcal abscess communities (SACs) within bone marrow or soft tissue adjacent to the primary site of infection [27,28]. SACs are surrounded by a pseudocapsule consisting of fibrin [29,30,31] which serves as a barrier for immune cells and antibiotics [32], allowing the SAC to survive within the host for many weeks [27,30,31,33]. Hence, it is evident why current antibiotic treatment regimens are not always effective for orthopaedic and device-related infections.

Enzybiotics are a general class of enzyme-based antimicrobials that can target components of the bacterial cell wall or biofilm matrix and present a potential alternative therapeutic approach for treating ODRI [34]. Certain Enzybiotics can degrade the bacterial cell wall as well as the surrounding biofilm matrix regardless of the bacterium’s metabolic state. Endolysins, the most prominent enzybiotics, are derived from bacteriophages. They generally possess hydrolase activity and function by cleaving various conserved chemical bonds within peptidoglycan, the primary component making up the cell wall of bacteria [35]. As cell wall integrity is essential to maintain the strong turgor pressure within the bacterial cell, cleavage of the peptidoglycan disrupts that integrity, leading to lysis and immediate death of the bacterium [34]. Similar in both structure and function to endolysins are certain bacteriocins, bactericidal proteins, or peptides produced by bacteria as defensive weapons [36]. Originally discovered in 1964 [37], lysostaphin is the most prominent and well-studied staphylococcal bacteriocin. It is an antimicrobial enzyme produced by strains of *S. simulans*, which recognizes structures unique to the staphylococcal genus [38]. Since certain enzybiotics target highly conserved structures within the bacterial cell wall, bacteria are unable to evolve resistance mechanisms, making them ideal for targeting antibiotic-resistant pathogens [39]. Previous research has also established that endolysins and certain bacteriocins feature antibiofilm properties [40,41,42,43], meaning they may hold greater promise for targeting established infections than conventional antibiotics. Bacteriophages can also express enzymes with specific antibiofilm activities. Recently, the polysaccharide depolymerase DA7, an enzyme that degrades poly-β-1,6-*N*-acetyl glucosamine (PNAG), a major biofilm component in staphylococci, was demonstrated to effectively disrupt *S. aureus* biofilms [44]. This protein was shown to have significant biofilm-clearing properties, and its effectiveness is synergistic with certain bactericidal endolysins [43,45].

Enzybiotics have shown promising efficacy both in vitro and in vivo [46]. The first example of a bacteriolytic enzyme being used to treat systemic infection was in 2002 in a mouse model of B. anthracis bacteremia [47]. This first example set off a wave of research involving the discovery and development of bacteriolytic enzymes for the rapid treatment of infection. In recent years, significant efforts have been made to develop enzybiotics, primarily endolysins, for certain clinical applications [48]. A recent systematic review detailing the discovery of endolysin, use in biotechnological applications, and development for clinical use provides a significant level of background [49]. Despite this, little has been explored regarding the potential of enzybiotics to treat established ODRIs in vivo. This may partly be due to the short half-life and efficient renal clearance of enzybiotics when administered systemically [34,50]. However, endolysins have been shown to be effective in treating certain local infections such as atopic dermatitis, where they performed better than standard antibiotic treatments in animals and in a human case study [51,52]. Endolysins are known to function synergistically with each other, as well as with classical antibiotics, portending their combined usage in future studies [34,53,54,55]. A recent study revealed the effectiveness of lysostaphin application via a hydrogel to prevent the onset of S. aureus ODRI [56]. In a separate study, the application of the endolysin PlySs2 was effective in combination with vancomycin in treating an in vivo model of staphylococcal prosthetic joint infection [57]. We, therefore, set out to explore whether an enzybiotic combination specifically tailored for its efficacy in vivo could be effective in treating established ODRI.

Here, we apply three enzybiotics: the chimeric lytic enzymes M23LST(L)_SH3b2638A (M23) and CHAPGH15_SH3bALE1 (GH15), as well as the DA7 polysaccharide depolymerase, as a combination regimen, and determine their effectiveness at eradicating established *S. aureus* infection. M23 and GH15 were previously selected in a large screen (>300 constructs) based on their high activity in human serum [58]. The engineered M23 protein contains the enzymatic domain of lysostaphin fused to the cell-wall-targeting domain from the 2638A bacteriophage-derived endolysin [59]. Similarly, the GH15 enzyme contains the enzymatic domain from the LysGH15 enzyme [60], enhanced with the targeting domain from the ALE-1 bacteriocin [61]. Because the catalytic domain of GH15 is derived from an endolysin, it is assumed that no known resistance mechanism to counter its catalytic function or recognition of peptidoglycan exist. In a separate study, these two enzymes were also shown to perform well in conditions mimicking the eukaryotic cytosolic and lysosomal environments and were effective at treating an in vivo *S. aureus* abscess model [62]. Given the efficacy of these enzymes individually and the established benefits of combining enzymes and antibiotics with varying mechanisms of action, we set out to evaluate the potential of this combined regimen, tailored to in vivo efficacy, biofilm activity, and low risk of resistance, in the treatment of ODRI in vitro and in vivo.

## 2. Materials and Methods

### 2.1. Bacterial Strains

Three *Staphylococcus aureus* strains were used in this study. EDCC 5443 (German Collection of Microorganisms and Cell Cultures GmbH) is a human pathogen that has multiple antibiotic resistances and was isolated from a human patient with implant problems. JAR 06.01.31 was isolated from a human patient with a periprosthetic knee infection (culture collection of Switzerland #890). USA300 AH-LAC is a methicillin-resistant *S. aureus* laboratory strain [63,64]. All strains were grown directly from frozen stocks in tryptic soy broth (TSB) at 37 °C. *E. coli* ClearColi BL21(DE3) (Lucigen, Middleton, WI, USA) was grown at 37 °C in Luria-Bertani (LB) medium (10 g/L tryptone, 5 g/L yeast extract, 8 g/L NaCl (pH 7.4)) and on LB agar (LB medium plus 14 g/L agar) supplemented with suitable antibiotics for maintenance of episomal expression vectors.

### 2.2. Production and Purification of Enzybiotics

The chimeric endolysins M23-LST_SH3b2638A and CHAPGH15_SH3bALE1 [65] were produced in *E. coli* and purified essentially as previously described [58,66]. In brief, plasmids encoding the desired constructs were transformed into *E. coli* ClearColi BL21(DE3) for endotoxin-free protein production. Cells were grown in LB-PE medium [67] supplemented with 100 µg/mL ampicillin and, once an OD600 of 0.5 was reached, protein production was induced by the addition of 0.5 M Isopropyl β-d-1-thiogalactopyranoside, followed by further incubation at 19 °C for 18 h. After the harvesting of cells and one freeze-thaw cycle, cells were disrupted by sonication (Bandelin Sonopuls HD 2076; 5 × 1 min, 1 s pulse/rest intervals, 80% power). Lysates were cleared by incubation with 5 units of DNase I (Thermo Fisher Scientific, Basel, Switzerland), centrifugation at 20,000× *g* for 1 h at 4 °C, and filtration (0.45 μm). Proteins were purified from the cleared crude extracts by cation exchange chromatography (CIEX), using a 5 mL HiTrap SP-FF on a fast protein liquid chromatography (FPLC) device (ÄKTA Purifier, GE Healthcare Life Sciences, Boston, MA, USA) with UV detection at 280 nm, as previously described [65]. The entire purification process was performed in an endotoxin-free environment at room temperature. Purified proteins were dialyzed into phosphate-buffered saline (PBS) and lyophilized in aliquots. Before lyophilization, endotoxin concentrations were determined using an EndoZyme kit (Hyglos, Regensburg, Germany) according to the manufacturer’s instructions, and protein identity and purity were checked by SDS-PAGE. Immediately before use, lyophilized proteins were reconstituted by the addition of deionized water.

The C-terminally 6× His-tagged polysaccharide depolymerase DA7 [43] was produced as described for the chimeric endolysins, with the following exceptions: pellets were resuspended in buffer A (50 mM Na_2_HPO_4_, 500 mM NaCl, 5 mM imidazole, pH 8.0) before cell disruption. Instead of CIEX, the protein was purified by immobilized metal ion affinity chromatography (IMAC) on the ÄKTA Purifier, using a 5 mL HisTrap FF Nickel Sepharose 6 Fast Flow column (Cytiva, Glattbrugg, Switzerland). Proteins were washed with buffer A and eluted by a linear gradient from 100% buffer A to 100% buffer B (50 mM Na_2_HPO_4_, 500 mM NaCl, 250 mM imidazole, pH 8.0), collecting fractions between 25% and 65% buffer B. Collected fractions were dialyzed against PBS and lyophilized. To test for protein identity and purity, 4 μg of protein was analyzed by SDS-PAGE using Mini-Protean TGX-stain-free precast gels (Bio-Rad, Hercules, CA, USA).

### 2.3. Cytotoxicity and Endotoxin Evaluation

Cytotoxicity assays were performed using the Pierce LDH cytotoxicity assay kit (Thermo Fisher Scientific, Waltham, MA, USA) according to the manufacturer’s protocol. Briefly, BJ-1 human fibroblasts were cultured in Dulbecco’s Modified Eagle Medium (DMEM) media (Gibco, Thermo Fisher Scientific, Basel, Switzerland) supplemented with glutamine, 1 g/L glucose, and 10% fetal bovine serum. A total of 2 × 10^6^ BJ-1 fibroblasts were treated with 5 µM GH15 and M23 and 1 µM DA7 polysaccharide depolymerase for 15 h, as described for overnight biofilm treatment experiments. The absorption of the supernatant was measured at 490 nm (signal) and 680 nm (background) with an automated spectrophotometer plate reader. After background subtraction, cytotoxicity was determined by dividing the signal of the samples by the signal of a maximum LDH-Activity. Maximum activity was obtained through lysis of the same number of cells using 0.5% Triton-X in PBS and collection of the resulting supernatant.

Endotoxin readouts for all three separate enzymes were performed using the Pierce chromogenic endotoxin quant kit, according to the manufacturer’s protocol. Five µM GH15 and M23, and 1 µM DA7 polysaccharide depolymerase were tested. Absorbance at 405 nm was measured using an automated spectrophotometer plate reader. Endotoxin readouts were expressed as relative absorbance units. Two standards (0.1 EU and 0.01 EU) provided by the manufacturer were utilized as control readouts and presented in the graph for comparison.

### 2.4. In Vitro Evaluations

#### 2.4.1. Planktonic Cell Assays

The turbidity reduction assay was performed as previously described, with minor modifications [58]. *S. aureus* USA300 AH-LAC was grown to log phase before being adjusted to an OD600 of 1.0 in PBS. Lysins were added to a final concentration of 100 nM, and OD600 measurements were taken every 20 min for 5 h in a 96-well format using an automated spectrophotometer plate reader to observe a reduction in optical density over time. Cells without lysins were also measured over time as a comparative control. PBS was used as a blank control. To evaluate CFU numbers after an overnight lysin treatment, *S. aureus* strains USA300 AH-LAC, JAR 06.01.31, and EDCC 5443 were prepared as described above in PBS. Subsequently, 100 µg/mL vancomycin or 1 µM of an equimolar ratio of M23-LST_SH3b2638A and CHAPGH15_SH3bALE1 were added, and bacteria were treated overnight. CFU numbers were evaluated by serially diluting the mixtures and plating on tryptic soy agar (TSA) plates and counting the resulting colonies, and expressed as CFU/mL of suspension.

#### 2.4.2. Peg Biofilm Assays

To evaluate the antibiofilm efficacy of enzybiotics, an MBEC Assay Biofilm Inoculator (Innovotech, Edmonton, AB, Canada) was used, consisting of a plate with 96 pegs that offer a surface for biofilms to grow on that sit submerged in the wells of a 96-well plate. To grow biofilms, a culture of the respective *S. aureus* strain was prepared and incubated overnight at 37 °C and diluted to 1:100 with fresh TSB supplemented with 0.25% (*m*/*v*) glucose and 1% human plasma (*v*/*v*). A sterile 96-well flat-bottom polystyrene plate was filled in each well with 200 μL of the diluted culture, the peg lid placed on top, and sealed with parafilm to prevent evaporation. The plate was incubated at 37 °C for 24 h with gentle tilting to allow for biofilm growth. After incubation, the peg lid was removed and washed once with PBS to remove free-floating bacteria. A fresh 96-well plate was prepared with 200 μL treatment solution per well. PBS served as a control treatment. Enzybiotic solutions were always prepared at a 1 μM concentration. Vancomycin treatments were provided at a concentration of 100 μg/mL and gentamicin at 150 μg/mL, diluted in PBS. Treatments proceeded for 24 h. To evaluate biofilm treatments, peg lids were removed, washed twice in PBS to remove residual treatment solutions, and placed in a fresh 96-well plate containing 200 μL in each well. The plate was sonicated partially submerged in a sonicating water bath (Bandelin electronic, Berlin, Germany) at 35 kHz for 30 min to remove the remaining biofilm from the pegs. CFU counts of the resulting solutions were evaluated by performing serial dilutions and plating 10 μL streaks onto TSA plates. CFU counts were reported as CFU/mL in the recovery solution. All groups were performed in technical triplicate per experiment.

#### 2.4.3. Titanium Device Biofilm Model

Biofilms were prepared similar to those described above. One cm-diameter titanium discs were produced in-house at the AO Research Institute workshop. The discs were sterilized by autoclaving and immersed in wells of a 48-well plate containing 300 μL of TSB medium supplemented with 0.25% (*m*/*v*) glucose and 1% human plasma (*v*/*v*) containing a 1:100 dilution of the respective *S. aureus* overnight culture. The plate containing titanium discs was incubated overnight at 37 °C without shaking to allow biofilms to grow on the metal surface. The following day, the discs were transferred to a fresh 48-well plate and washed twice with PBS to remove residual suspended bacteria. Three hundred μL of treatment solutions (prepared as described above for the peg model) were applied to each well and incubated overnight without shaking. To quantify the remaining biofilm, the discs were washed in PBS and placed in a small glass jar filled with 1 mL PBS. Throughout this process, the discs were kept in the same orientation in order to keep the biofilm exposed and because less biofilm developed on the bottom of the discs. These jars were submerged in a sonicating water bath and sonicated at 35 kHz for 5 min to remove the remaining biofilm. The solution containing recovered bacteria was serially diluted as described above and plated on TSA plates. CFU counts were reported as total recovered CFU counts per disc. All groups were performed in technical triplicate per experiment.

#### 2.4.4. In Vitro SAC Model

In vitro SACs were generated, exposed to treatments, and processed for CFU quantification as described previously [32]. Briefly, in vitro SACs were generated in a 24-well Transwell system (polyester membrane with a porosity of 0.4 µm; Corning Life Sciences B.V., Amsterdam, the Netherlands) by layering 25 µL bacterial solution containing approximately 14 CFUs of *S. aureus* JAR 06.01.31 in between two collagen gel layers, prepared from rat collagen type I solution (1.78 mg/mL, pH 7.4; Gibco, Basel, Switzerland) by following manufacturer’s instructions. The gel with *S. aureus* JAR 06.01.31 was supplemented with in total 400 µL pooled human plasma (Regional Blood Donation Service SRK Graubünden, Chur, Switzerland). Following overnight incubation, the human plasma layer was removed from the collagen gel containing mature in vitro SACs. The SACs were then either challenged with PBS, gentamicin, and vancomycin-containing PBS, PBS containing enzybiotics, or the combination of the antibiotic gentamicin and vancomycin with enzybiotics in PBS. Treatment solutions were prepared as described above for the peg model and were applied on top of the collagen gel with in vitro SACs and into the well underlying the Transwell; 200 µL and 600 µL, respectively. The in vitro SACs were challenged for 24 h. Bacterial numbers were quantified by first removing any liquids from on top of the in vitro SAC-containing collagen gel, washing the samples three times with 500 µL PBS for 5 min, and transferring the samples into self-standing 50 mL tubes containing 1 mm zirconium oxide beads (Next Advance, Troy, NY, USA) and 250 µL PBS. Homogenization of the samples was performed with the Bullet Blender (Next Advance) for 3 min (speed 10) and sonication for 3 min at 35 kHz. Afterwards, 10-fold serial dilutions were prepared of the samples, which were pipetted in 10 µL streaks onto TSA plates and incubated overnight at 37 °C. Values were reported as CFU counts per sample. All groups were performed in technical duplicate or triplicate per experiment.

The LIVE/DEAD^®^ BacLight Bacterial Viability Kit (Invitrogen, Basel, Switzerland) was used to visualize bacteria within in vitro SACs by applying 500 µL of the staining solution, including Syto9 and propidium iodide (PI). Stained samples were imaged with a Zeiss LSM 800 confocal laser scanning microscope (Zeiss, Oberkochen, Germany), and image processing was performed with the ZEN (blue edition) software (Zeiss, Oberkochen, Germany).

#### 2.4.5. Scanning Electron Microscopy

In vitro, SAC samples for scanning electron microscopy (SEM) were fixed with McDowell’s fixative, dehydrated with an ascending ethanol series, and paraffin-embedded. The paraffin-embedded samples were then sectioned, deparaffinized, air-dried, and placed with the glass slide onto a specimen stub. Biofilm samples grown on titanium discs were dehydrated by submerging in an ascending ethanol series: 50%, 60%, 70%, 80%, 90%, 96%, and 100% ethanol for 5 min each. Samples were sputter-coated with 10 nm gold/palladium (80:20) using a BAL-TEC MED 020 (BAL- TEC AG, Pfaeffikon, Switzerland). Scanning electron microscopy (SEM: Hitachi FESEM 4700; Hitachi, Tokyo, Japan) was performed on representative samples with a secondary electron (SE) and yttrium aluminum garnet (YAG) backscattered electron (BSE) detector (Hitachi, Tokyo, Japan) and analyzed with digital acquisition software Quartz PCI (Quartz Imaging Corporation, Vancouver, BC, Canada).

### 2.5. In Vivo Observations

#### 2.5.1. Murine Model of Fracture-Related Infection

The animal study was approved by the ethical committee of the canton of Graubünden in Switzerland (approval number 12_2020 and 22E:2020) and was carried out in a research facility accredited by the Association for Assessment and Accreditation for Laboratory Animal Care (AAALAC) International.

Mice underwent an initial operation to create a femoral osteotomy, which was repaired with a 4-hole titanium MouseFix plate with analgesia and anesthesia as previously described [68]. At the time of surgery, mice were inoculated with 10^4^ CFU of SA strain USA300 at the logarithmic growth phase. The wound was closed and allowed to mature for 5 days before revision surgery and debridement was performed, and treatment was administered for the subsequent 5 days. After a 3-day washout period, mice were euthanized, and the bone, plate, and surrounding soft tissue were evaluated by CFU counting.

Female C57BL/6 mice (20–28 weeks old) were utilized for the study, with three mice from each group designated for histopathological analysis and nine for quantitative bacteriology. Animals were group-housed under a 12 h light/dark regimen in individually ventilated cages (XJ, Allentown) and fed with standard chow (3436, Provimi Kliba, Kaiseraugst, Switzerland). Mice were acclimatized for at least 2 weeks to the housing conditions. Behaviorally incompatible mice were rehoused together with different mice.

Revision surgery was performed five days after the initial osteotomy and inoculation surgery. The initial approach was performed identically as described above. After opening the infected leg, a small amount of infected tissue was cut away and sampled for bacterial analysis. The osteotomy was flushed with 1 mL sterile saline, which was subsequently collected for bacterial quantification to confirm infection. Mice treated with enzybiotics were delivered 50 µL equimolar enzybiotic combination (M23/GH15/DA7 at 1 mg/mL). The wound was then closed in the same manner as after the initial surgery. Postoperative analgesia was continued until five days after the revision surgery with tramadol in the drinking water. For subsequent daily treatments, mice were put under general anesthesia using Sevoflurane as an anesthetic agent (as described above). During this 5-day treatment period, mice received one of four daily treatment regimens (Table 1).

Mice were euthanized on day 13. After putting the animals in Sevoflurane anesthesia, the animals were euthanized by means of cervical dislocation and exsanguination. A macroscopic examination of the external body surface, all orifices, and surgery sites was conducted on all animals. A total of ten mice were euthanized early during the study for a variety of reasons. These reasons included a high scoring (high level of sedentary or aggravated behavior), hyperthermia during CT scanning, the presence of an open wound, broken femur, or a high weight loss. These mice were replaced with reserves and operated on in a subsequent surgical round in order to maintain adequate numbers in each group.

For quantitative bacteriology, femur, implant, and the soft tissue surrounding the repaired osteotomy were removed, weighed, and placed in 1 mL of room temperature sterile PBS. Bone and tissues were homogenized (Omni TH, tissue homogenizer TH-02/TH21649, Kennesaw, GA, USA) in 1 mL of PBS. The implant was sonicated for 2 min to dislodge attached bacteria. Sonicated implant fluid and tissue homogenates were serially diluted, plated on blood agar (BA) plates, and incubated overnight at 37 °C. Bacterial colonies were counted, and the resulting numbers were presented as CFUs per gram of tissue or CFU per sonicated implant. To confirm that colonies were *S. aureus*, random colonies were picked and tested using the StaphLatex agglutination test (Thermo Fisher Scientific, Waltham, MA, USA).

#### 2.5.2. Computed Tomography

The operated leg was scanned using computer tomography directly after inoculation surgery, after revision surgery, and post-mortem. Bone volume within and around the defect was monitored using the cone-beam in vivo microCT scanner VivaCT40 (SCANCO Medical AG, Wangen-Brüttisellen, Switzerland). Animals were scanned under sevoflurane anesthesia immediately following surgery, at 5 days post-operatively, and after euthanasia on day 13. A region of 2.2 mm, centered on the defect, was scanned with a 21.5 mm field of view, at a voltage of 70 kV and 114 μA current, 300 ms integration time, and 1000 projections per scan. The projections were then reconstructed across an image matrix of 2048 × 2048 pixels with an isotropic voxel size of 10.5 μm. All image analysis was performed using Amira software (Amira version 6.3, FEI Company, Hillsboro, OR, USA).

The postoperative scans served as baseline scans to define two regions of interest (ROI). The osteotomy gap itself was segmented via interpolation between both osteotomy lines to determine the initial gap size, and the bone outside of the defect site including an additional 0.42 mm on either side of the osteotomy region. Subsequent scans were registered to the baseline scans using rigid registration. With the resulting transformation matrix, the ROI were transformed to the respective scans to allow evaluation of the same regions. Prior to evaluation the scans were gaussian-filtered (standard deviation = 2, kernel size = 3) to remove noise. Bone was segmented automatically (threshold = 650 mgCaHA/mL), and bone volume was computed within the ROIs using direct voxel counting methods. All image processing and analysis were performed with custom scripts in Amira.

#### 2.5.3. Histology

Skin and fur were removed from mouse upper hind legs and fixed in 4% buffered formalin for >1 week. Sample preparation, fixation, Brown and Brenn and, H&E stains of histological sections were performed as previously described [69].

### 2.6. Statistics

Statistical analyses were performed in GraphPad Prism 8.1.0 (GraphPad Software, San Diego, CA, USA). For experiments that compared only two groups, unpaired Student’s *t*-tests were utilized to evaluate the statistical significance. For experiments comparing multiple groups with three different *S. aureus* strains, ordinary two-way ANOVAs were performed with Dunnett’s multiple comparison test. For SAC treatment, where a single *S. aureus* strain was tested, an ordinary one-way ANOVA was performed with Dunnett’s multiple comparison test. To evaluate the statistical significance in the animal CFU data, Kruskal-Wallis tests with Dunn’s multiple comparison tests were performed after testing for normal data distribution. In all cases, a *p*-value < 0.05 was considered significant.

## 3. Results

### 3.1. Purified Enzybiotics Demonstrate Rapid Bacterial Killing as Well as Antibiofilm Activity

**Figure 1:**
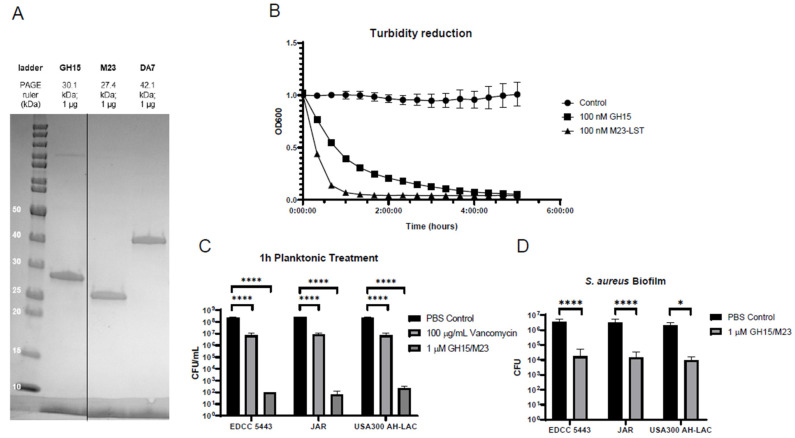
Peptidoglycan hydrolases (lysins) are potent antimicrobial enzymes. (**A**) SDS-PAGE analysis of the three indicated enzybiotics. A molecular weight ladder is in the left lane and allows for a molecular weight comparison. (**B**) A turbidity reduction assay of OD600 1.0 cultures of *S. aureus* USA300 with 100 nM of the two indicated lysins added. The control curve contained bacteria only (*n* = 3). (**C**) Comparison of CFU counts following a 1-h treatment of an OD600 1.0 culture of the three indicated *S. aureus* strains with vancomycin or the two lysins (*n* = 3). (**D**) Measurement of biofilm eradication using a 96-well peg lid model of the three indicated *S. aureus* strains after the indicated treatments (*n* = 5). * *p* ≤ 0.05, **** *p* ≤ 0.0001.

The GH15, M23, and DA7 depolymerase were expressed and produced in *Escherichia coli* via episomal vectors and purified using liquid chromatography. SDS-PAGE analysis of the extracted proteins was performed to evaluate their purity and visualize any major contaminants. As seen in Figure 1A, the gel exhibits only single bands at the expected molecular weights of the respective constructs, signifying good purity. Furthermore, the enzymes were determined to be clear of endotoxins, as well as non-cytotoxic to cultured fibroblasts (Supplementary Materials Figure S1). To evaluate the effectiveness of the two lysins GH15 and M23 against planktonic SA, a turbidity reduction assay was performed using a low concentration (100 nM) of each enzyme against a SA culture at the logarithmic growth phase. As would be expected for these two lysins, they rapidly cleared a turbid culture in a matter of hours, with the M23 enzyme acting more rapidly than GH15 (Figure 1B). The lysin combination was compared against a bactericidal concentration of vancomycin, the standard-of-care treatment for MRSA infections. We tested this against planktonic cultures of three clinical strains. All three strains demonstrated an equal level of susceptibility to both vancomycin and an equimolar mixture of the two lysins over a 1-h treatment period, with the lysins being vastly superior to vancomycin in their ability to quickly kill planktonic cells (Figure 1C). Finally, a peg biofilm model was utilized to evaluate the effectiveness of the two lysins at reducing bacterial numbers within SA biofilms. An overnight treatment of 24-h biofilms with the same 1 µM lysin combination demonstrated the ability to reduce over 99% of bacteria embedded in the biofilm in all three SA strains tested, compared to treatment with a PBS control (Figure 1D). Together, this suggested that the M23 and GH15 proteins are highly effective at reducing SA biofilms, in addition to featuring a highly rapid killing efficacy.

### 3.2. Addition of the DA7 Polysaccharide Depolymerase and Antibiotics Enhances Antibiofilm Activity

**Figure 2:**
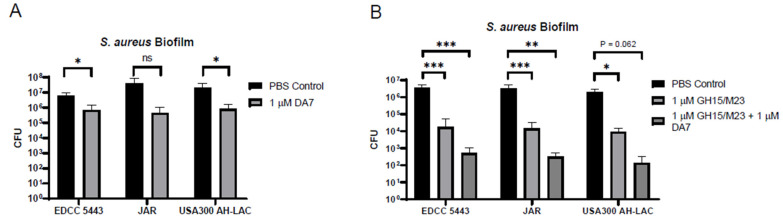
The DA7 polysaccharide depolymerase enhances the antibiofilm effects of peptidoglycan hydrolases (lysins). (**A**) Measurement of biofilm eradication using a 96-well peg lid model of the three indicated *S. aureus* strains after treatment with DA7 polysaccharide depolymerase (*n* = 3). (**B**) Biofilm eradication as in (**A**) comparing lysins (GH15 and M23) to lysins combined with the DA7 depolymerase (*n* = 5 for control and lysins; *n* = 3 for lysins + depolymerase). *** *p* ≤ 0.001, ** *p* ≤ 0.01, * *p* ≤ 0.05, ns: *p* > 0.05.

In an attempt to further improve upon our enzyme-based antibiofilm treatment regimen, we explored the use of the staphylococcal bacteriophage-derived depolymerase DA7. DA7 is known to target poly-β-1,6-N-acetyl glucosamine (PNAG), a major constituent of staphylococcal biofilms, and we hypothesize that its antibiofilm activities could thus enhance the bactericidal effects of the M23/GH15 combination. To test this, we quantified the CFU counts of SA biofilms treated with 1 µM DA7 depolymerase. Alone, this enzyme was able to reduce CFU numbers from recovered biofilms by more than 90% relative to a PBS control (Figure 2A) for all three tested SA strains. Combining the effects of the DA7 depolymerase with the bactericidal effects of the two lysins demonstrated an enhanced killing efficacy (Figure 2B).

**Figure 3:**
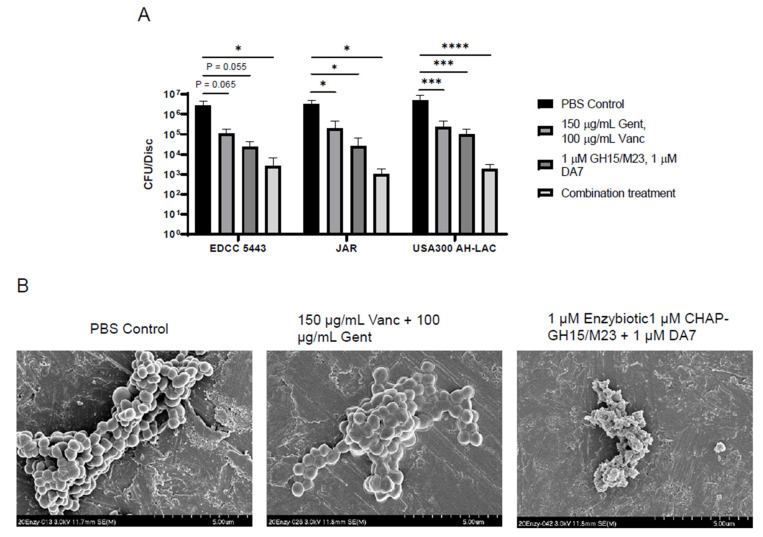
Enzybiotics and classical antimicrobial chemotherapy together enhance biofilm clearance in a titanium device biofilm model. (**A**) CFU counts recovered from biofilm of the three indicated *S. aureus* strains were grown on titanium discs, treated with enzybiotics, antibiotics. Combination treatment indicates a full treatment with 150 μg/mL Gent, 100 μg/mL Vanc, 1 μM equimolar GH15/M23/DA7. CFU counts recovered per disc are reported (*n* > 3). (**B**) Scanning electron microscopy (SEM) of treated biofilms grown on titanium discs. Scale bars are indicated at the bottom right of each image. **** *p* ≤ 0.0001, *** *p* ≤ 0.001, * *p* ≤ 0.05, ns: *p* > 0.05.

Bacterial lysins have been shown in several cases to function synergistically with classical antibiotic chemotherapy [34]. We, therefore, sought to determine whether our enzybiotic regimen containing the two lysins as well as the DA7 depolymerase could be enhanced by combining with antibiotics to treat SA biofilms. The enzybiotic regimen in combination with vancomycin did not demonstrate any additive antibiofilm effect for any of the three tested strains (Supplementary Figure S2). We believe this is due to vancomycin’s overall lack of antibiofilm activity. We, therefore, combined this regimen with gentamicin, which is the most commonly used antibiotic in local antibiotic delivery in orthopaedic trauma surgery and has been shown to be effective at removing staphylococcal biofilms from surfaces [70]. This full combination treatment was used against staphylococcal biofilms on a titanium disc model. As predicted, the addition of gentamicin and vancomycin together to the enzybiotic regimen showed enhanced antibiofilm activity, which was superior to the enzybiotic or antibiotic treatments alone (Figure 3A). We utilized scanning electron microscopy to directly observe enzybiotic-treated USA300 AH-LAC biofilms. Enzybiotic application showed a significant reduction in biofilm mass, as well as a significant effect on the integrity of the bacterial cells (Figure 3B). As expected, antibiotic-treated biofilms did not show the same level of physical clearance but likely contained many dead cells.

### 3.3. Enzybiotics Are Highly Effective at Targeting Staphylococcal Abscess Communities In Vitro

**Figure 4:**
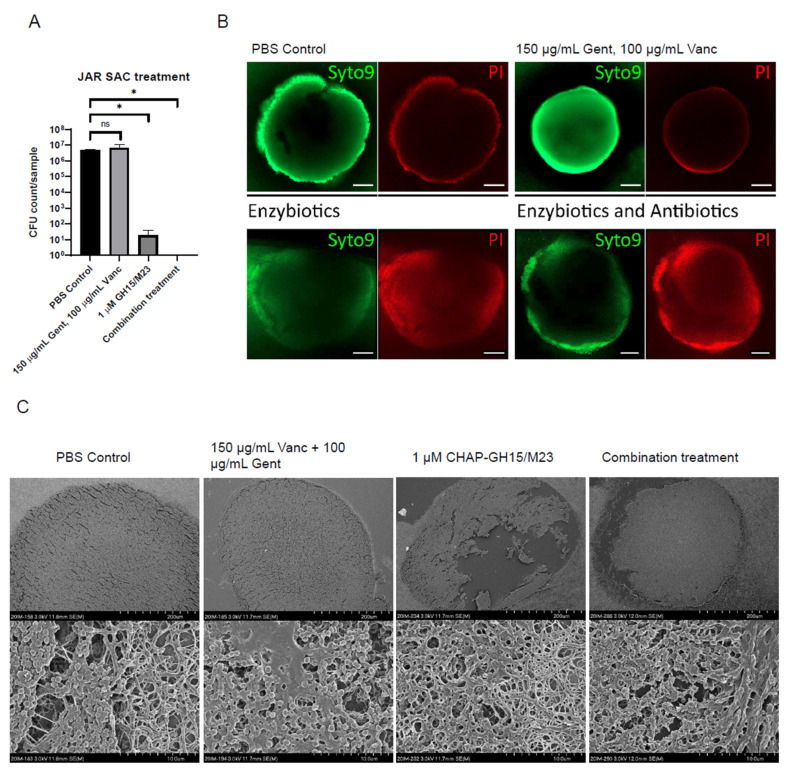
Enzybiotics are effective at treating an in vitro staphylococcal abscess communities (SAC) model. (**A**) Resulting CFU counts per sample of 24-h SACs treated with enzybiotics and antibiotics. Combination treatment indicates a full treatment with 150 μg/mL Gent, 100 μg/mL Vanc, 1 μM equimolar CHAP-GH15/M23 (*n* = 3). (**B**) Representative fluorescence microscopy images of treated SACs as in (**A**) stained with nucleic acid dyes Syto9 (green, membrane-permeable) and propidium iodide (red, non-membrane-permeable). Scale bars are 100 µm. (**C**) SEM images of treated SACs as in (**A**). Overview images are in the top panel, and magnified images are in the bottom panel. Scale bars are indicated at the bottom of each image. * *p* ≤ 0.05, ns = not significant.

Using an in vitro SAC model previously established in our lab [32], we evaluated the effectiveness of an equimolar M23/GH15 lysin combination in targeting SA cells growing within SACs. The equimolar GH15/M23 treatment alone or as combination therapy with antibiotics showed itself to be extremely effective at reducing SA CFU numbers in treated SACs, while the vancomycin and gentamicin together showed no measurable effect compared to the PBS control (Figure 4A). We further scrutinized these findings by performing a live/dead stain of the SACs with the nucleic acid dyes Syto9 (all cells: green) and propidium iodide (PI, dead cells: red). Whereas the PBS-treated and antibiotic-treated SACs only stained positive for PI at the periphery of the SACs, the enzybiotic and combination-treated SACs had a more diffuse PI signal throughout the entire SAC and also showed a more diffuse Syto9 signal (Figure 4B). Using SEM, we observed significant disturbances in SAC integrity (Figure 4C, upper). Only a few SA cells from enzybiotic- or combination-treated SACs appeared intact, and the residual SACs appeared to contain primarily the fibrin and collagen scaffolds, signifying that most SA had been fully lysed (Figure 4C, lower).

### 3.4. Enzybiotics Are Effective at Treating ODRI In Vivo

**Figure 5:**
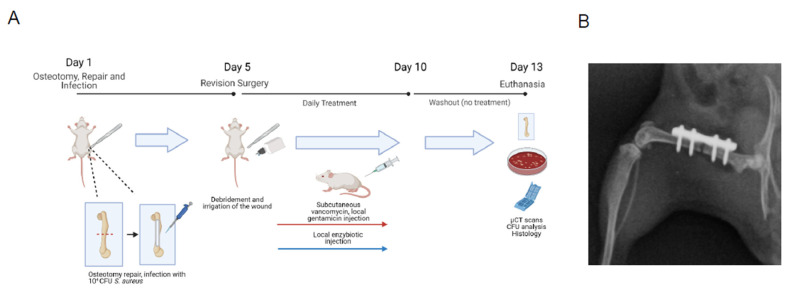

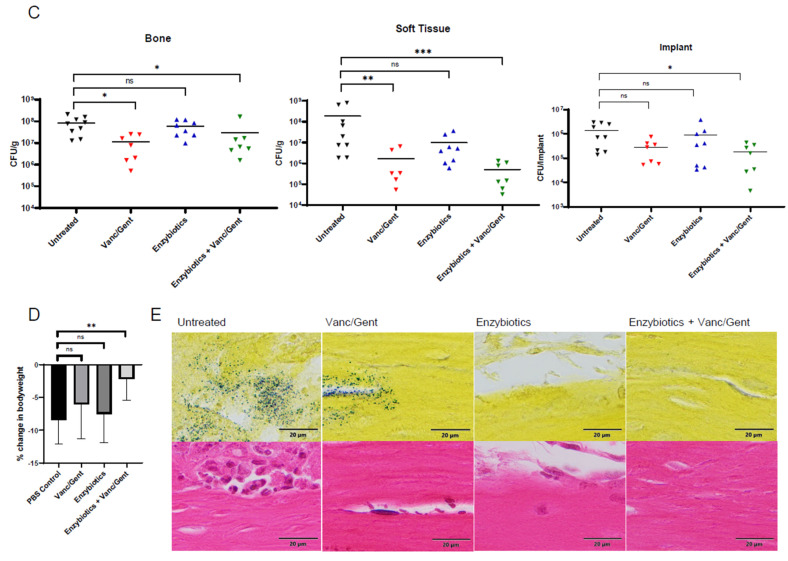
Enzybiotics are effective at treating MRSA fracture-related infection in combination with antibiotics in vivo. (**A**) An overview of the mouse model of fracture-related infection. On day 1, mice receive an osteotomy in the right femur, which is repaired with a 4-hole titanium plate. 10^4^ CFU of *S. aureus* USA300 is added directly on top of the plate before the wound is sutured closed. The infection is allowed to mature for 5 days before revision surgery is performed and initial treatment (antibiotics, enzybiotics, or both) is administered. Daily treatment proceeds for the subsequent five days before being halted for a three-day washout period. Mice are euthanized on day 13, and CFU counts of the femur, implant, and surrounding soft tissue are analyzed. (**B**) An example radiograph image of a mouse hindleg after receiving an osteotomy and repair surgery. (**C**) CFU counts from the homogenized femur (bone), surrounding soft tissue, and implant from mice receiving the indicated treatments. (**D**) Percent change in body weight from the time of initial surgery (day 0) to the time of euthanasia (day 13). (**E**) Representative Brown and Brenn (upper; SA in blue) and Hematoxylin and Eosin (lower) stains of mouse soft tissue sections adjacent to the osteotomy. The two stains were performed on adjacent histological sections from the same mouse receiving the indicated treatment regimen. * *p* ≤ 0.05, ** *p* ≤ 0.01, *** *p* ≤ 0.001, ns: *p* > 0.05.

To observe whether this enzybiotic regimen can treat an established infection, a mouse model of MRSA ODRI was devised that would allow for a sustained treatment period. An overview of this multi-stage in vivo infection treatment model is seen represented in Figure 5A, and a radiograph of the osteotomy repair can be seen in Figure 5B. Overall, enzybiotics alone did not significantly reduce overall CFU levels but did so in combination with antibiotics (gentamicin and vancomycin) relative to untreated mice (Figure 5C). Enzybiotic treatment did not show any treatment effect for the infected bone (Figure 5C, left). However, in the surrounding soft tissue, enzybiotics slightly decreased CFU levels relative to antibiotic treatment (Figure 5C, middle). Interestingly, the enzybiotic/antibiotic combination was the only treatment able to significantly reduce CFU counts on the infected device, likely due to the antibiofilm activities of the enzybiotic combination (Figure 5B, right). Weight loss of the mice over the course of the 13-day treatment period was measured, and we found that only the full enzybiotic/antibiotic treatment was able to significantly prevent weight loss relative to untreated mice (Figure 5D), while enzybiotics or antibiotics alone were unable to exert a significant effect on this outcome. Brown and Brenn staining of histological sections of soft tissue adjacent to the infected osteotomy generally showed less positive staining for SA after enzybiotic treatment relative to sections from antibiotic-treated or untreated mice (Figure 5E). This local CFU reduction conferred by the enzybiotic/antibiotic combination led to a measurable but statistically insignificant decrease in the loss of bone volume around the infected osteotomy at the time due to infection (Supplementary Figure S3). Together, these data clearly demonstrate that in this in vivo model of acute MRSA infection, enzybiotics in combination with classical antibiotics can improve soft tissue treatment and reduce bacterial numbers on an implant.

## 4. Discussion

New strategies for targeting biofilms may greatly increase success rates for the treatment of *S. aureus* ODRI while decreasing recurrent infection rates. Surgical interventions, in combination with conventional antibiotic agents, remain the most effective means for treating these types of infections at the present time. Rapidly acting antibiofilm and antimicrobial agents that can be applied locally as an injection or during debridement are highly desirable [71]. Specifically, antimicrobials that can target EPS components and persister cells and rapidly kill bacteria in a manner independent of the bacterial metabolism should theoretically provide a better alternative to the current standard for treating orthopaedic infection. Classical antibiotics have a disadvantage in that they do not directly affect the biofilm matrix, meaning that their diffusion into a biofilm is sometimes limited. Circumventing this problem, enzybiotics enzymatically break down cellular and matrix material, targeting cells regardless of their antibiotic resistance profile. Currently known as one of the fastest-acting antimicrobials, the use of endolysins to target antibiotic-resistant *S. aureus* has been extensively explored in the laboratory, but lesser so in the clinic [34,50]. Most pertinently, and in contrast to bacteriophages from which they are derived, no endolysin resistance mechanism is known to exist [34]. Hence, we set out to explore the use of a combination of enzybiotics for the treatment of ODRI.

Here we have shown that an enzybiotic combination is highly effective at clearing both biofilms and SACs in vitro, and in combination with antibiotics, is effective at treating severe and difficult-to-treat infection in vivo. Initially, we evaluated the additive effects of different components in eradicating biofilm. The DA7 depolymerase is on its own reasonably effective at reducing CFU numbers from a biofilm, presumably due to its degradation of EPS biofilm components, which causes bacteria to fall off the biofilm surface [43]. Combined with the M23 and GH15 lysins, the DA7 enhanced overall antibiofilm effects. We speculate this is because the DA7 increases the accessibility of the bacterial cell wall to the lysins by degrading the biofilm matrix, allowing bacteria to be killed more effectively by the lysins. Enzybiotics are large macromolecules and, therefore, suffer from lower diffusion rates compared to small-molecule antibiotics. Indeed, endolysins that do not possess a cell-wall binding domain perform slightly better than their full-length counterparts in plate lysis or overlay assays, which require diffusion through a semi-solid matrix, presumably due to their smaller size and lower cell-wall affinity [72]. Encoded by the *icaADBD* operon, PNAG is frequently found in staphylococcal biofilms and is normally the primary exopolysaccharide [73,74]. However, in an infection, several other components can make up the matrix, such as host proteins, DNA, teichoic acids, and other extracellular polysaccharides. While degradation of PNAG clearly added supplemental effects in our in vitro setup, the addition of other degradative enzymes such as DNAses and fibrinolytic enzymes, which have been shown to have certain antibiofilm effects, could perhaps show increased benefits [75,76,77].

USA300 strains are the most prevalent cause of acute MRSA infections in the USA [78]. Of these, roughly 85% present as abscesses [78]. Most cases are relatively minor, but some can be life-threatening. SACs are considered to be a common feature of localized staphylococcal infections and are accompanied by localized neutrophil infiltration [33]. During SAC development, the abscess acquires certain defining features, including a dense pocket of live, metabolically inactive bacteria within a fibrin pseudocapsule, viable and necrotic neutrophils, and some macrophages [30,33,79,80]. It is considered to be a mechanism adapted by the host to contain and eliminate the invading pathogen. However, if the abscess is allowed to mature, it also can serve as a mode of persistence for *S. aureus* [29]. Our enzybiotic combination showed itself to be highly superior to classical antibiotics at reducing CFU numbers in an in vitro SAC model. Bacteria residing in an in vitro SAC are surrounded by a fibrin pseudocapsule similar to in vivo SACs [32]. Previously, it was shown that the fibrin pseudocapsule could severely limit antibiotics from reaching bacteria within a SAC and the antibiotic gentamicin had minimal antibacterial activity that was restricted to the outer layer of bacteria within the SAC [32]. In contrast, the enzybiotics enter the in vitro SACs and begin lysing bacteria throughout the entire SAC structure, and since they actively lyse bacteria, can facilitate their own diffusion by breaking down bacterial cell wall and debris. In our experiments, Syto9 and PI stains showed a more diffuse signal after SACs were treated with enzybiotics, suggesting that SA cells had been lysed and the stained debris was still somewhat contained within the pseudocapsule. Overall, our in vitro result is in agreement with the recent finding that lysins can be effective at treating an in vivo subcutaneous abscess model [62].

A few animal studies evaluating the abilities of several staphylococcal endolysins to clear infection have been performed to date. In one of the most relevant examples, all mice infected intra-peritoneally with MRSA and immediately injected with a solution containing several individual endolysins recovered, and the effect was identical to the vancomycin-treated control group [81]. Intravitreal injection of the Ply187 endolysin has been shown to protect mice from developing *S. aureus* endophthalmitis by reducing CFU numbers, reducing inflammatory cytokines, and neutrophil infiltration [82]. A phase II clinical trial is currently in the recruitment process to treat *S. aureus* bacteremia with the SAL200 endolysin (identifier: NCT03089697). A second anti-*S. aureus* endolysin, CF-301, was recently evaluated in a phase I trial, which observed no serious AEs following intravenous injection, and was found to be generally well-tolerated [83]. This endolysin (ContraFect Corporation) recently showed in a phase II trial improved clinical outcomes in bacteremia/right-sided endocarditis relative to standard antibiotics (identifier: NCT03163446) and is moving into a phase III trial (identifier: NCT04160468), which includes testing for treatment of persistent MRSA infection following COVID-19 (identifier: NCT04597242). This endolysin treatment, however, only showed a significant effect in the MRSA-infected subgroup, but not overall. Thus, enzyme-based antimicrobials hold significant promise for use in the clinic in combination with standard-of-care therapy, where such standard treatments alone have clear shortcomings.

Enzybiotic-coated devices have been used successfully to prevent *S. aureus* infection in a murine model [84]. In one prominent study, researchers used lysostaphin to prevent ODRI by adding the enzyme to a PEG-based hydrogel that adheres to exposed tissue [56]. Lysostaphin encapsulation within the hydrogel showed enhanced enzyme stability and allowed for controlled release of the enzyme. The system performed better than prophylactic antibiotic therapy in preventing the onset of MRSA infection while promoting bone repair and restoring a sterile inflammatory environment required for bone healing. The same researchers later added BMP-2 to the same treatment in a murine radial segmental defect infection model, whereupon they observed additional bone regeneration complementing the successful infection prophylaxis [85] However, the ability to treat established infection in vivo was not evaluated, despite the established knowledge that lysostaphin can adequately disrupt biofilms [86,87]. This is an important distinction, as infection treatment usually requires the clearance of biofilms and is generally far more difficult than infection prevention. Moreover, unlike endolysins, *S. aureus* can acquire resistance to bacteriocins [88,89] and would perhaps be better suited in combination with an endolysin. In fact, our M23 enzyme is a lysostaphin derivative, and strains could become resistant to its effects during the infection process. However, we theorize that co-application of the GH15 enzyme, which is an endolysin, could subvert this issue as no endolysin resistance mechanisms are known to exist. Additionally, enzyme application via a hydrogel does present an attractive option for many reasons, especially in cases of surgical application. Therefore, future studies will examine whether any biocompatible hydrogels can be used to apply enzybiotics, and whether the slow-release properties they offer would benefit infection treatment with enzybiotics.

Enzybiotics have certain drawbacks. For example, because they cause the lysis of the bacterial cell, they can promote the release of intracellular toxins that could be harmful to the host. Because enzybiotics are large molecules, they also suffer from poor diffusion rates. In our infected mouse model, we observed that CFU numbers were significantly reduced in combination with classical antibiotics in both the soft tissue and on the implanted device, clearly demonstrating that enzybiotics may enhance treatment efficacies together with classical treatment protocols. However, they did not show a significant effect in the infected femur, although this was also the case with the antibiotic treatment. We assume this to be due to poor diffusion into the bone, as is the case for many other antibiotics, especially when applied locally [90]. One possible way to circumvent this would be to apply enzybiotics systemically, although this faces its own hurdles. A recent study using the PlySs2 endolysin against an in vivo model of prosthetic joint infection found little significant reduction in CFU numbers in the infected bone following intraperitoneal injection of the lysin, demonstrating that systemic administration does not necessarily ameliorate bone treatment [57]. Systemic lysin administration has always faced the hurdle of rapid renal clearance. For example, the serum half-life of the pneumococcal Cpl-1 lysin is approximately 20 min [91]. This fact is the primary reason we chose to apply our enzybiotic combination as a daily local injection. Many strategies are currently being explored that would allow extending the time lysins can remain active in the bloodstream [58], strategies we hope would add more potential to the unique benefits offered by bacteriolytic and antibiofilm enzymes and other enzybiotics.

## 5. Conclusions

In this study, we have shown that the M23 and CHAP-GH15 enzymes are highly effective at rapidly killing planktonic SA. Further, they are highly effective at reducing SA biofilm, but this effect is enhanced with the addition of the DA7 polysaccharide depolymerase. Antibiofilm activity can be further enhanced when a gentamicin/vancomycin treatment is added, although this effect is likely, not due to the vancomycin but to the more bioactive gentamicin. In an in vitro SAC model, enzybitotics are vastly superior to classical antibiotics at breaching the pseudocapsule and eradicating the dense SA community inside. In a mouse model of ODRI, enzybiotics clearly enhance treatment outcomes and supplement the effects of classical antibiotics by reducing CFU numbers in the soft tissue and on the infected implant. An enzybiotic combination regimen may hold promise for the treatment of severe ODRI and warrants further investigation.

## Figures and Tables

**Table 1:** Treatment regimens for 5 days following revision surgery.

	Number of Mice Survived until Day 13 and Evaluated	Treatment	Additional Vancomycin Treatment	Frequency
Untreated	9 for bacteriology 3 for CT/Histo	50 µL sterile saline	none	1× per day directly into infected soft tissue
Enzybiotics	8 for bacteriology 3 for CT/Histo	50 µL equimolar enzybiotics (M23, GH15, DA7). 1 mg/mL total enzyme concentration	none	1× per day directly into infected soft tissue
Enzybiotics + Vanc/Gent	7 for bacteriology 2 for CT/Histo	50 µL equimolar enzybiotics (M23, GH15, DA7). 1 mg/mL total enzyme concentration; supplemented with 200 µg gentamicin	110 mg/kg delivered subcutaneously	Enzybiotics/gentamicin 1× per day directly into infected soft tissue vancomycin 2× per day
Vanc/Gent	7 for bacteriology 2 for CT/Histo	50 µL saline containing 200 µg gentamicin	110 mg/kg delivered subcutaneously	Gentamicin 1× per day directly into infected soft tissue vancomycin 2× per day

## Data Availability

The data presented in this study are available on request from the corresponding author.

## Supplementary Materials

The following are available online at https://www.mdpi.com/article/10.3390/antibiotics10101186/s1, Figure S1: Enzybiotics are neither cytotoxic nor endotoxic; Figure S2: Vancomycin alone does not supplement the antibiofilm effects of enzybiotics; Figure S3: Computed tomography scanning of osteotomy and measurement of bone volume change over time.
