# Supplementary material for: An Enzybiotic Regimen for the Treatment of Methicillin-Resistant Staphylococcus aureus Orthopaedic Device-Related Infection
Source: Antibiotics (Basel). 2021 Sep 29;10(10):1186. doi: 10.3390/antibiotics10101186 (PMC8533017; doi:10.3390/antibiotics10101186)
Supplement: Supplementary file 1 [file antibiotics-10-01186-s001.zip › antibiotics-1302535-supplementary.pdf]

Supplementary Materials

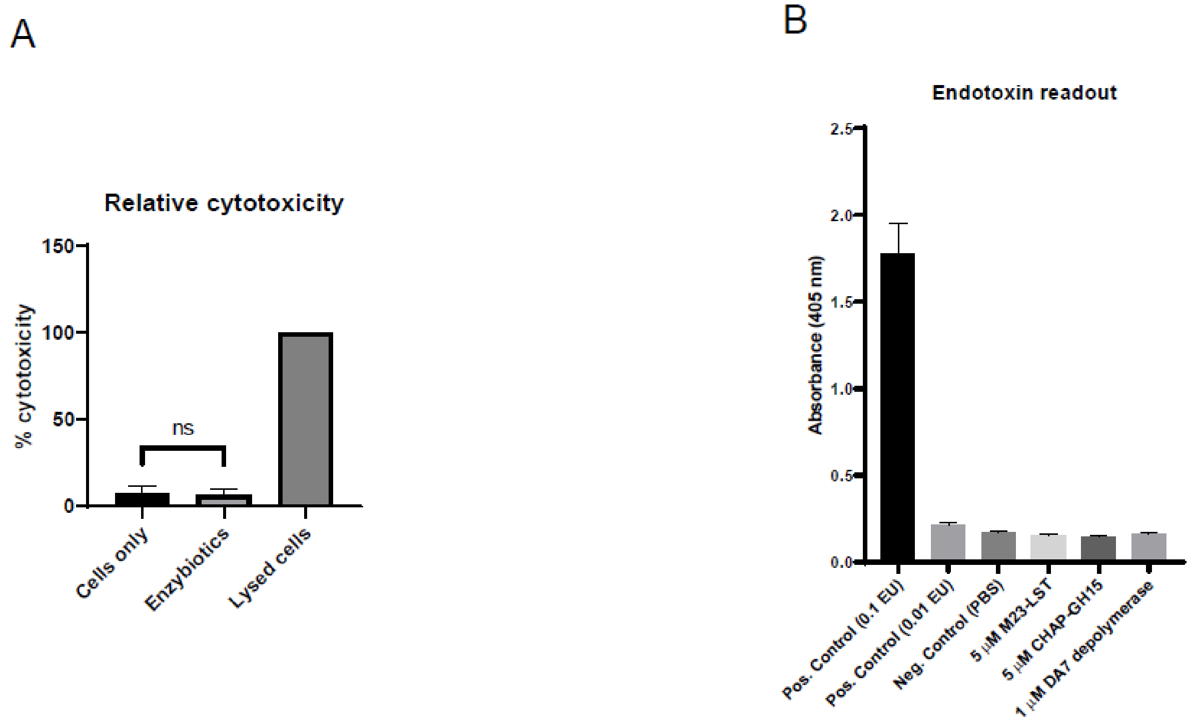

Figure S1. Enzybiotics are neither cytotoxic nor endotoxic. A) Relative cytotoxicity readouts of cultured BJ-1 fibroblasts treated with a mixture of 5 µM GH15/M23 and 1 µM DA7 depolymerase. A readout from cells only indicates untreated cells, while lysed cells serve as a positive control "maximum" readout value; ns = not significant. **B**) An endotoxin readout of each individual enzybiotic at the indicated concentrations. The highest and lowest standard values are provided as positive and negative controls.

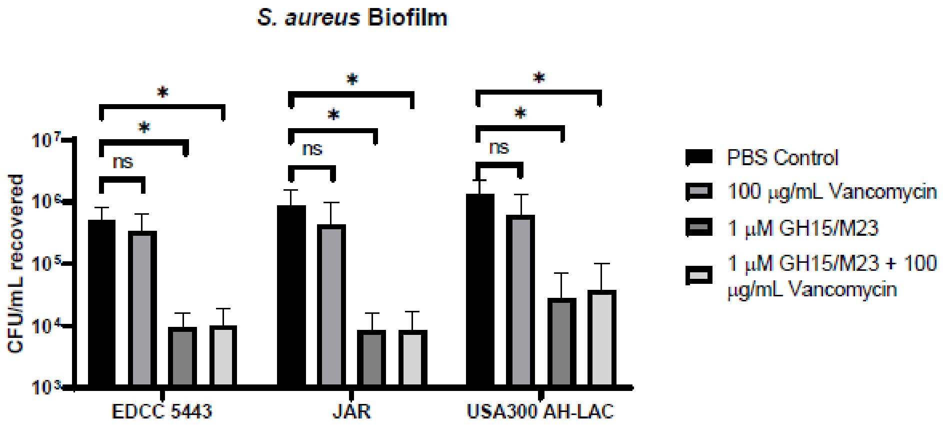

Figure S2. Vancomycin alone does not supplement the antibiofilm effects of enzybiotics. Measurement of biofilm eradication using a 96-well peg lid model of the three indicated *S. aureus* strains after treatments with vancomycin, enzybiotics or both. * $p \leq 0.05$, ns = $p > 0.05$.

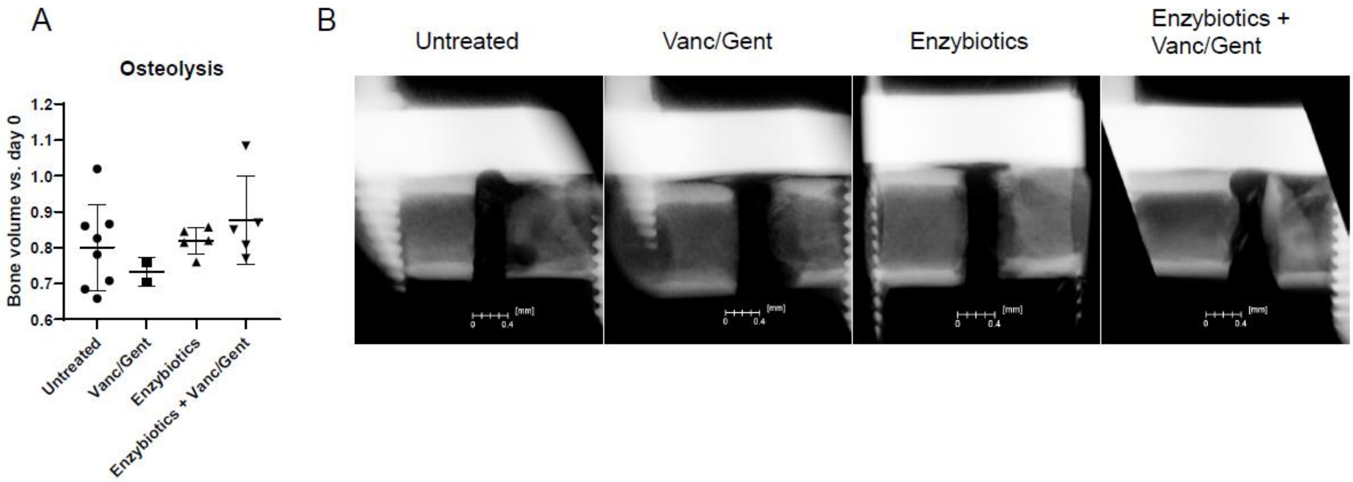

Figure S3. Computed tomography scanning of osteotomy and measurement of bone volume change over time. A) Relative bone volumes around the infected bone defect after treatments relative to bone volumes at the time of initial surgery, as measured by CT imaging. **B**) Representative digitally reconstructed radiographs from the CT images of the bone defect for each indicated treatment group at final endpoint (following treatment). Scale bars in mm are provided to compare the space inside of the defect.
